# Biocompatible oil core nanocapsules as potential co-carriers of paclitaxel and fluorescent markers: preparation, characterization, and bioimaging

**DOI:** 10.1007/s00396-015-3767-5

**Published:** 2015-09-18

**Authors:** Sławomir Drozdek, Urszula Bazylińska

**Affiliations:** Departament of Organic and Pharmaceutical Technology, Faculty of Chemistry, Wroclaw University of Technology, Wybrzeże Wyspiańskiego 27, 50-370 Wrocław, Poland

**Keywords:** Polyester nanocarriers, Cytostatic drug, Nile Red, Coumarin-6, Colloidal stability, Breast cancer cells, Cellular internalization

## Abstract

The present work is focused on the long-term stability and in vitro cellular internalization of newly designed biocompatible polyester nanocapsules prepared via nanoprecipitation approach with mean diameter <165 nm and narrow size distribution, dedicated to theranostic applications. We monitored the optical, morphological, and biological properties of the nanocarriers loaded by multifunctional cargo, i.e., paclitaxel (PTX) and a fluorescent marker: coumarin-6 (CR-6) or Nile Red (NR), by fluorescence and UV–vis spectroscopy (encapsulation efficiency), dynamic light scattering (average size expressed as hydrodynamic diameter, *D*_H_), zeta potential (*ζ*, colloidal stability), atomic force microscopy (AFM, imaging), and confocal laser scanning microscopy (CLSM, nanocapsule visualization, and cellular internalization in vitro by human breast cancer MCF-7/WT cells). The fabricated nanocapsules with optimal composition of oleic phase, i.e., coconut oil, palm oil, and Capmul MCM, as well as polymeric shell, i.e., polylactic acid (PLA), poly (*ε*-caprolactone) (PCL), and poly (lactide-co-glycolide) (PLGA), showed high loading capacity, long-term stability, and improved localization of the active cargo in studied tumor cells. Therefore, our results prove that the studied polyester oil core nanocapsules provide lifelong and biocompatible nanocarriers suitable for in vivo administration and for diagnostic applications.

## Introduction

Since, in the past decade, major progress in the development, synthesis, design, and purposeful application of various types of nanocontainers has been achieved, polymers play an important role in the stability of colloidal dispersions [1, 2], while polymeric nanoparticles have been extensively studied in the pharmaceutical and biotechnological field as bioactive agent carriers that are suitable for delivering a drug to the injured site [3, 4]. Among the different drug delivery approaches (e.g., microemulsions, functional micelles, dendrimers, nanospheres), nanocapsules have attracted increased attention [5–7]. They have advantages over conventional drug delivery systems, as they can increase the bioavailability, solubility, and sustained release of many potent drugs which are otherwise difficult to deliver orally [8, 9]. Therefore, poorly water-soluble cytostatic drugs, including naturally occurring taxoles, can be used in many encapsulation technologies to improve their several favorable biological properties, such as nontoxicity, dissolution performance, and biocompatibility by means of template-mediated and self-assembly processes [1, 9, 10]. Additionally, due to the high loading capacity of interiors, nanocapsules could allow simultaneous multicargo encapsulation, i.e., a hydrophobic drug (not showing fluorescence) and a fluorescent marker (organic dye or quantum dot), creating multifunctional nanocarriers dedicated for theranostic applications [11, 12]. Furthermore, nanocapsules can be found in specific drug delivery system, as they can penetrate the cell membrane and increase its permeability for many potent drugs which are otherwise difficult to deliver to the target tissues [13, 14].

Correspondingly, in each nanocarrier preparation method, special importance has to be placed on the aspects determining the container features responsible for the best attainment of the final goal. For example, if one mainly needs to provide high long-term stability of the colloidal system and to protect it from aggregation, either charged or sterically branched container shells are necessary [8, 15]. Nanoprecipitation technique—also called solvent displacement or interfacial deposition—is the most applicable method for polymeric nanocapsules preparation. Nanoprecipitation is based on spontaneous emulsification of the organic internal phase with the dissolved polymer and oil (in the case of nanocapsules fabrication) into the aqueous external phase in the presence of a surfactant [16]. Commonly, as a straightforward and quick methodology, nanoprecipitation does not require high shearing/stirring rates, sonication, or very high temperatures, and often, it enables the production of small nanoparticles (100–200 nm) with narrow unimodal distribution and exhibiting a high drug loading capacity and long-term stability [8]. Additionally, it has been successfully applied in the encapsulation of different hydrophobic drugs and other bioactive molecules [8, 17].

The polymeric nanocapsule shell generally consists of hydrophilic polymer while their core commonly employs oil dissolving the drug. Therefore, the application of the appropriate biodegradable oleic phase (consisting of monoglycerides and diglycerides or vegetable oils) is also very important for achieving the high loading capacity and physical stability of the multifunctional cargo [8, 18, 19]. Furthermore, an ideal drug delivery system apart from its nanoscale size, high loading capacity of active molecules, and long-term stability should also have high biocompatibility to enhance the active cargo bioactivity as well as to reduce its side effects [2]. Therefore, efforts to produce pure and highly biocompatible polymers have allowed scientists to apply them in several scientific areas including tissue engineering and drug delivery [14]. Biocompatible polyesters containing biodegradable natural units as polylactic acid (PLA), poly (*ε*-caprolactone) (PCL), and poly (lactide-co-glycolide) (PLGA) have emerged as a fascinating class of biomedical materials. The polyester bonds are sufficiently stable in the blood circulation and extracellular fluid. But, after cell internalization, they will be cleaved rapidly under intracellular reductive conditions [20]. The pursuit of nanocarriers based on bioreducible oils and polymers forms the basis of a new strategy for drug release and cellular internalization.

As the continuation of our regular studies [8, 15, 18, 19, 21–23] on the new drug delivery nanosystems, we aim the present contribution at the design and characterization of biocompatible nanocarriers employed for effective co-encapsulation of a cytostatic drug paclitaxel (PTX) and a fluorescent marker (coumarin-6 (CR-6) or Nile Red (NR)). The present study is focused on the comparison of the drug delivery properties of polyester-based, i.e., PLA, PCL, or PLGA nanocapsules, with different oleic phase, i.e., coconut oil, palm oil, and monodiglyceride of medium-chain fatty acids (Capmul MCM), prepared according to the interfacial precipitation technique, in terms of their physicochemical characteristics, colloidal stability, and their ability to improve cellular delivery and bioimaging of CR-6 or NR co-encapsulated with PTX in the tumor breast MCF-7/WT cells.

## Materials and methods

### Materials

PTX, NR, CR-6, PCL (Mw ∼14,000 Da), poly (d,l-lactide) (PLA, Mw ∼75,000–120,000 Da), PLGA (L/G ∼50/50, Mw ∼25,000 Da), palm oil, coconut oil, tetrahydrofuran (THF), and dimethylformamide (DMF) were obtained from Sigma-Aldrich. Capmul MCM C10 (glyceryl monocaprate) was a kind gift from ABITEC Corporation of USA. Cremophor EL^®^ (polyethoxylated castor oil) was obtained from BASF. All materials were used as received without further purification. Water used for all experiments was doubly distilled and purified by means of a Millipore (Bedford, MA) Milli-Q purification system.

### Fabrication of PLA, PCL, and PLGA nanocapsules with different oil core

Nanocapsules were fabricated by a direct solvent displacement (also called nanoprecipitation) method proposed by our group and described previously [8]. Briefly, the organic solution was consisted of PLA, PCL, or PLGA (5 mg/ml); coconut oil; palm oil; or Capmul MCM C10 (0.1 mg/ml) as oil phases and PTX (0.125 mg/ml), NR (0.076 mg/ml), or CR-6 (0.06 mg/ml) dissolved in tetrahydrofuran (THF). A constant volume (1 mL) of this organic phase was added dropwise under vigorous magnetic stirring to 5 ml of an aqueous solution containing Cremophor EL in a concentration of 1 % (*w*/*w*), which was maintained for 1 h. After this time, the organic solvent was evaporated at reduced pressure in a rotary evaporator (Büchi Rotavapor R-200) and nanocapsules were collected overnight. In the next step, the respective suspensions were separated via nanofiltration (using PES membranes with a pore size of 0.2 μm) and finally stored at 4 °C in the dark (*t* = 40 days). As a control, blank nanocapsules without PTX and the fluorescent markers were prepared in a similar manner.

### Nanocapsule characterization

#### Size and polydispersity measurements

The size distribution (i.e., the hydrodynamic diameter, *D*_H_) and polydispersity indexes (PdI) of the obtained nanocapsules were determined by dynamic light scattering (DLS) using Zetasizer Nano Series from Malvern Instruments with the detection angle of 173° in optically homogeneous square polystyrene cells. All measurements were performed at 25 °C. Each value was obtained as an average of three runs with at least ten measurements. The DTS (Nano) program was applied for data evaluation.

#### Zeta potential measurements

The zeta potential (*ζ*-potential) of nanocapsules suspended in 0.25 % (*w*/*v*) saline solution (pH 7.4, conductivity 2.0 mS/cm) was exemplarily measured by the microelectrophoretic method using a Malvern Zetasizer Nano ZS apparatus according to our previous studies [8]. Each value was obtained as an average of three subsequent runs of the instrument with at least 40 measurements.

#### Atomic force microscopy

The morphology of the nanocapsules was examined using the Veeco NanoScope Dimension V atomic force microscopy (AFM) with an RT ESP Veeco tube scanner. The scanning speed was 0.5 Hz and a low-resonance-frequency pyramidal silicon cantilever resonating at 250–331 kHz was employed (at a constant force of 20–80 N/m). The amplitude of the resonance was set manually to the lowest possible amplitude for stable imaging within the contamination layer present on the surface. Before observations, the nanocapsules were allowed to adsorb on a freshly cleaved mica surface for 12 h by dipping it in the suspension. Then, the excess of substrate was removed by rinsing the mica plates in double-distilled water for 1 min and drying at room temperature.

#### Encapsulation efficiency

To confirm the encapsulation of PTX, NR, and CR-6, UV–vis spectroscopy was applied. The UV–vis absorbance measurements were performed using a Metertech SP8001 spectrophotometer with 1-cm pathlength thermostated quartz cell, and the drug concentration was calculated using the calibration plot. Encapsulation efficiency was determined according to the adapted method previously described [24]. All measurements were performed in triplicate.

#### Optical measurements

Fluorescence emission spectra of the both free and encapsulated NR or CR-6 molecules were recorded by a Hitachi F-4500 spectrofluorometer with a constant scan speed (240 nm*/*min). The excitation and emission slit widths were 10 nm. All samples were measured in quartz cell of 1-cm optical path length at room temperature.

#### Fluorescence microscopy

The samples were examined under the confocal scanning laser microscope (the ZEISS Cell Observer SD Spinning Disk Confocal Microscope, Germany). All nanocapsules were diluted in water before observation. NR emission was observed by using a 559-nm excitation filter (*λ*_Em_ = 636 nm), whereas CR-6 was excited using a 456-nm laser line (*λ*_Em_ = 500 nm).

#### Cell culture

The studies were performed on a well-examined human malignant doxorubicin-sensitive cell (MCF-7/WT) line. The cell line for biological experiments was a kind gift from the Department of Tumor Biology, Comprehensive Center of Oncology, Maria Sklodowska-Curie Memorial Institute, Gliwice Branch, Poland. Cells were grown in cultured DMEM (Sigma) supplemented with 10 % fetal calf serum (BioWhittaker) and antibiotics (penicillin/streptomycin, Sigma) in 25-ml TC flasks (Falcon) at 37 °C, in 5 % CO_2_ humidified atmosphere according to our previous studies [15, 25]. For the experiments, the cells were removed by trypsinization (trypsin 0.025 % and EDTA 0.02 %; Sigma) and rinsed twice with phosphate-buffered saline (PBS).

#### Cellular internalization of loaded nanocarriers

Intracellular localization, accumulation, and distribution of PTX delivered to the breast cancer MCF-7/WT cells with nanocapsules were analyzed by confocal laser scanning microscopy (CLSM). After cultivation in standard conditions, the MCF-7/WT cells were trypsinized from the culture flasks and harvested on cover glasses in Petri dishes for 24 h. In the next step, free PTX, CR-6, or NR was added to reach the final concentration of 1 × 10^−6^ M in control positive cell sample. The experimental cell samples were treated with multifunctional cargo-loaded nanocapsules at appropriate doses to attain an equivalent concentration of the active cargo. Additional control cells contained polymeric nanocarrier analogs without dyes and PTX molecules. Thereafter, the cells were incubated for 24 h at 37 °C, fixed with 4 % paraformaldehyde, and washed with PBS. The slides were then mounted on the stage of a CLSM 510 Meta confocal laser scanning microscope (Carl Zeiss GmbH, Jena, Germany). The fluorescent emission of CR-6 or NR delivered to the cells was determined after excitation at 405 or 488 nm. The images (512 × 512 pixels) were recorded by employing a Plan Apochromat 63× oil immersion objective (NA 1.4). An HFT 405/488/561 dichroic mirror and an LP 575 emission filter were used in channel 2 for detecting fluorescence marker emission.

## Results and discussion

### Optimization, characterization, and stability of nanocapsules loaded with PTX and fluorescent markers

**Fig. 1:**
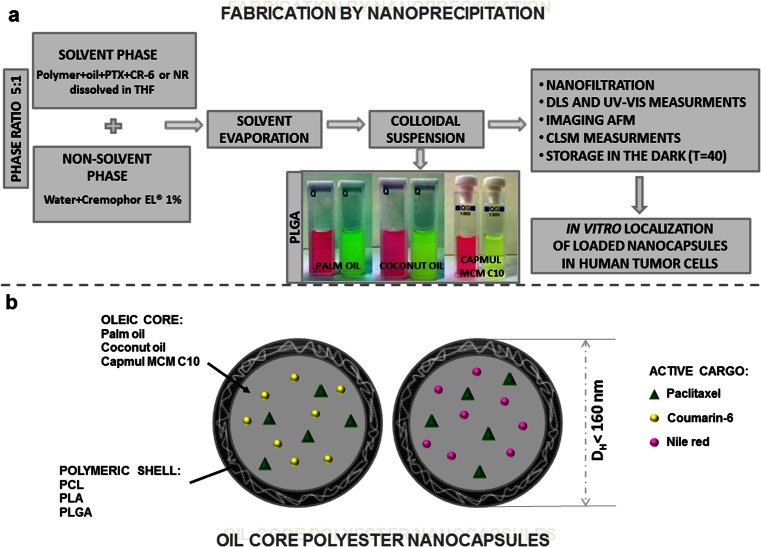
**a** The illustrative scheme of the fabrication and characterization of polyester nanocapsules loaded by paclitaxel (PTX) and fluorescent markers (NR and CR-6). **b** The main components of the obtained nanocarriers

Polymeric nanocapsules for co-encapsulation of PTX and fluorescent markers (NR or CR-6) were prepared by the nanoprecipitation method [8] using polyesters PLGA, PLA, and PCL as biodegradable and biocompatible polymeric components; Cremophor EL^®^ as nonionic surfactant for stabilization of nanoparticle dispersion; and palm oil, coconut oil, or glyceryl monocaprate (Capmul MCM C10) as biocompatible oil phases (see Fig. 1). The structures of compounds used for the formation of nanocapsules with their abbreviations are collected in Table 1. Nanoprecipitation is an extensively used method for the preparation of nanocarriers with therapeutic agents and/or diagnostic compounds embedded in the hydrophobic core and/or polymeric matrices. This method allows for rapid access to nanospheres or nanocapsules in large quantity and easy scale up in pharmaceutical industry [16]. The appropriate concentrations of polymers, surfactant, and the ratio of organic to aqueous phase (1:5) were selected according to our previous studies [8], whereas the other compound concentrations were determined experimentally.

**Table 1:**
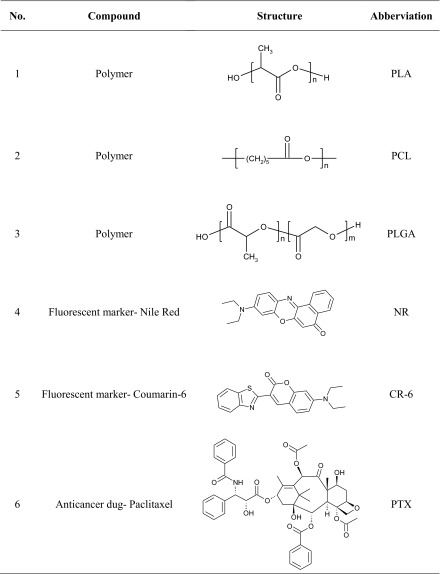
Chemical structures and abbreviations of chosen compounds

In our studies, PTX-CR-6- and PTX-NR-loaded nanocarriers were produced and characterized in terms of mean diameter (*D*_H_), polydispersity index (PdI), and zeta potential (*ζ*) for 40 days to assess the stability of these systems during storage. The physicochemical characterization of nanocapsules and PTX, CR-6, and NR encapsulation efficiencies are summarized in Table 2. PTX-CR-6- and PTX-NR-loaded nanocapsules and empty ones with a mean diameter between 95 and 164 nm, low PdI <0.2, and slightly negative zeta potential values between −1 and −6 mV were fabricated. The obtained mean diameters are smaller than the diameters of the nanoparticles loaded with PTX-NR obtained previously [26] and fulfill the requirements of literature to appear to be appropriated for bioimaging cancer cells and for drug delivery system by the occurrence of enhanced permeability and retention (EPR) effect. The EPR effect in solid tumors is one of the few tumor-specific characteristics that become a standard in antitumor drug delivery. However, most solid tumors have blood vessels with defective architecture, such as large tight junctions between endothelial cells varying in size from 100 to 780 nm and lack of smooth muscle layers, so that macromolecules will have the opportunity to escape from tumor blood vessels and accumulate selectively in tumor tissues, whereas they could not cross normal blood capillaries, as the vascular endothelium in most healthy tissues has a pore size of 2 nm, and 6-nm pores are observed in postcapillary venules, which will result in less side effects [27]. Furthermore, the proper size and surface properties of the nanocarriers play an important role in drug-efficient encapsulation and release, in cellular uptake, and in its pharmacokinetics and biodistribution. Nanoparticles could also reduce the drug resistance of some cancer cells [12]. As it has been proved, nanoparticles ≥200 nm induce the host systems cells and they can be taken up by the Kupffer cells faster than their smaller counterparts, resulting in their rapid clearance from circulation system. Nanocarriers with size ≤150 nm can leak through fenestration of the vascular endothelium, while nanostructures with diameter <10 and <30 nm are definitely cleared through the kidney or lymph node, respectively [28, 29]. Though it is still not totally clear which nanoparticle size leads toward highest therapeutic efficacy in vivo and the most beneficial biodistribution, when nanoparticles are systemically administered, there is a universal agreement that nanoparticles should be lower than 200 nm.

**Table 2:** Characteristics of polymeric nanocarriers (*T* = 0 and *T* = 40 days)

		*T* = 0													*T* = 40					
System	Oil core	Nile Red					Coumarin-6					Empty			Nile Red		Coumarin-6		Empty	
		*D*_H_ (nm)	PdI	*ζ* (mV)	EE_PTX_ (%)	EE_NR_ (%)	*D*_H_ (nm)	PdI	*ζ* (mV)	EE_PTX_ (%)	EE_CR6_ (%)	*D*_H_ (nm)	PdI	*ζ* (mV)	*D*_H_ (nm)	PdI	*D*_H_ (nm)	PdI	*D*_H_ (nm)	PdI
	PLA																			
1a	Palm oil	135 ± 7	0.15 ± 0.01	−3 ± 1	52 ± 3	65 ± 3	121 ± 6	0.19 ± 0.01	−2 ± 1	54 ± 3	70 ± 3	138 ± 8	0.19 ± 0.01	−2 ± 1	143 ± 7	0.16 ± 0.01	123 ± 6	0.19 ± 0.01	138 ± 7	0.19 ± 0.01
1b	Coconut oil	113 ± 5	0.16 ± 0.01	−2 ± 1	60 ± 3	55 ± 2	115 ± 4	0.13 ± 0.01	−1 ± 1	58 ± 3	72 ± 3	107 ± 4	0.18 ± 0.01	−1 ± 1	116 ± 5	0.18 ± 0.01	114 ± 5	0.18 ± 0.01	109 ± 5	0.18 ± 0.01
1c	Capmul MCM 10	104 ± 5	0.19 ± 0.01	−2 ± 1	80 ± 4	89 ± 4	99 ± 4	0.19 ± 0.01	−1 ± 1	70 ± 3	75 ± 3	112 ± 6	0.19 ± 0.01	−1 ± 1	103 ± 5	0.19 ± 0.01	116 ± 5	0.19 ± 0.01	112 ± 5	0.20 ± 0.02
	PCL																			
2a	Palm oil	164 ± 9	0.11 ± 0.01	−4 ± 1	62 ± 3	73 ± 3	156 ± 8	0.10 ± 0.01	−5 ± 1	55 ± 3	67 ± 3	160 ± 9	0.13 ± 0.01	−3 ± 1	168 ± 9	0.13 ± 0.01	165 ± 8	0.13 ± 0.01	164 ± 8	0.14 ± 0.01
2b	Coconut oil	112 ± 5	0.14 ± 0.01	−4 ± 1	75 ± 3	71 ± 3	110 ± 5	0.16 ± 0.01	−4 ± 1	69 ± 3	58 ± 2	112 ± 7	0.17 ± 0.01	−3 ± 1	109 ± 5	0.15 ± 0.01	118 ± 5	0.16 ± 0.01	114 ± 5	0.18 ± 0.01
2c	Capmul MCM 10	106 ± 5	0.16 ± 0.01	−3 ± 1	75 ± 3	81 ± 4	103 ± 4	0.15 ± 0.01	−3 ± 1	71 ± 3	78 ± 3	111 ± 4	0.16 ± 0.01	−4 ± 1	108 ± 5	0.16 ± 0.01	113 ± 5	0.16 ± 0.01	116 ± 6	0.17 ± 0.01
	PLGA																			
3a	Palm oil	95 ± 4	0.20 ± 0.01	−5 ± 1	47 ± 3	51 ± 2	135 ± 7	0.13 ± 0.01	−6 ± 1	72 ± 3	81 ± 4	140 ± 6	0.14 ± 0.01	−5 ± 1	101 ± 4	0.20 ± 0.02	141 ± 7	0.16 ± 0.02	106 ± 5	0.14 ± 0.01
3b	Coconut oil	123 ± 6	0.16 ± 0.01	−3 ± 1	78 ± 3	69 ± 3	109 ± 5	0.15 ± 0.01	−2 ± 1	70 ± 3	72 ± 3	112 ± 7	0.15 ± 0.01	−5 ± 1	126 ± 7	0.17 ± 0.01	111 ± 5	0.17 ± 0.01	116 ± 6	0.17 ± 0.01
3c	Capmul MCM 10	104 ± 5	0.16 ± 0.01	−3 ± 1	73 ± 3	85 ± 4	103 ± 5	0.15 ± 0.01	−5 ± 1	76 ± 3	84 ± 4	105 ± 5	0.15 ± 0.01	−4 ± 1	101 ± 4	0.17 ± 0.01	105 ± 5	0.17 ± 0.01	109 ± 5	0.16 ± 0.01

*D*_*H*_ hydrodynamic diameter, *PdI* polydispersity index, *ζ* zeta potential, *EE*_*PTX*_ encapsulation efficiency of PTX, *EE*_*NR*_ encapsulation efficiency of NR, *EE*_*CR6*_ encapsulation efficiency of CR-6

**Fig. 2:**
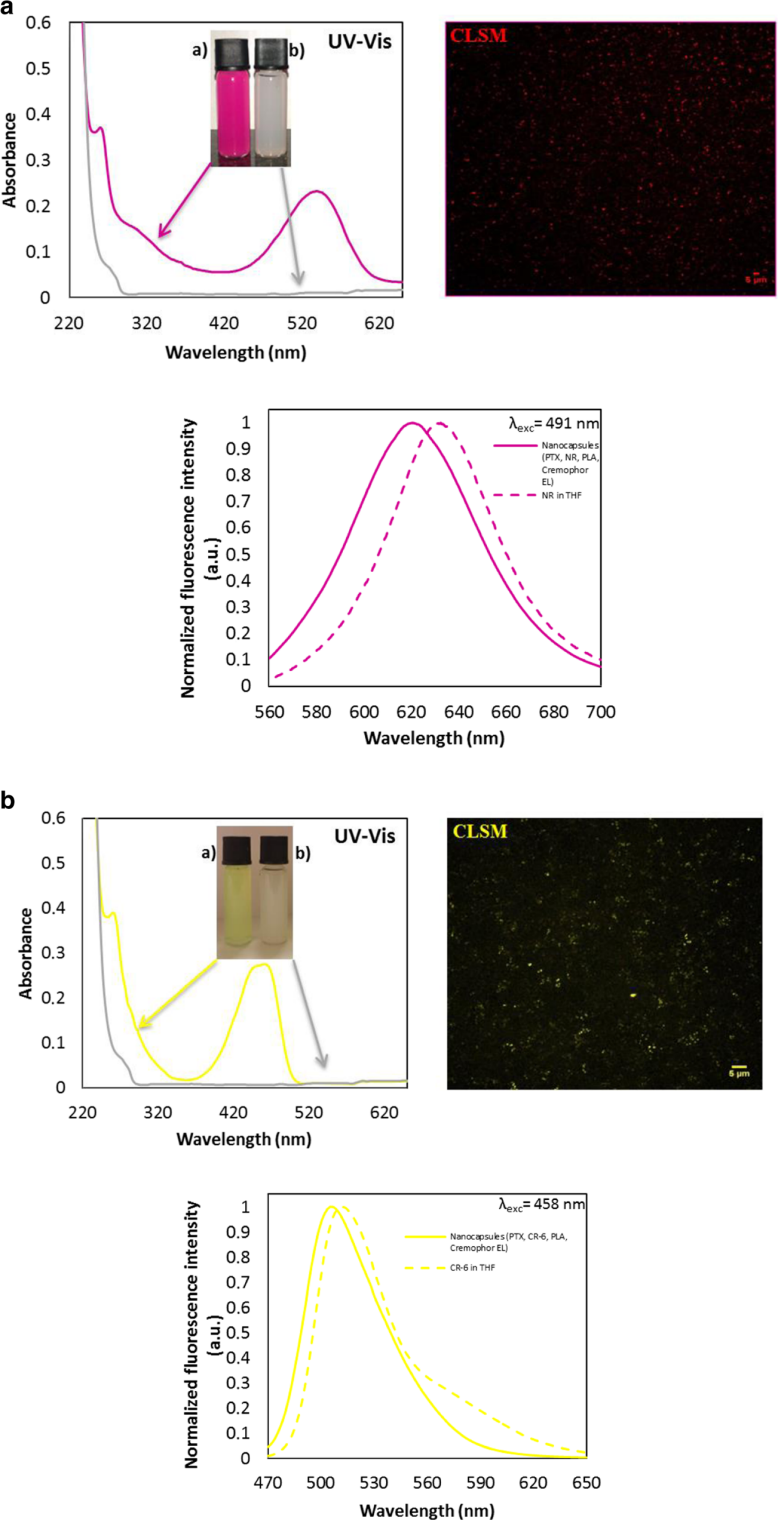
**a** Room temperature absorption spectra of PTX- and NR-loaded Cremophor EL/PLA/Capmul MCM 10 nanocapsules compared to the empty nanocarriers as well as fluorescence microscopy (CLSM) image of the nanocapsules (*right side*) and emission spectra of encapsulated NR compared to the dye molecules dissolved in THF (*below*). **b** Room temperature absorption spectra of PTX- and CR-6-loaded Cremophor EL/PLA/Capmul MCM 10 nanocapsules compared to the empty nanocarriers as well as fluorescence microscopy (CLSM) image of the loaded nanocarriers (*right side*) and emission spectra of encapsulated CR-6 compared to the dye molecules dissolved in THF (*below*)

The UV–vis spectroscopy was used to demonstrate the encapsulation of PTX and fluorescent markers in oil core nanocapsules, which technique corresponds well with data from other reports for the nanocarriers loaded by the same active cargo [10, 24]. Figure 2a, b shows the UV–vis spectra of PTX-NR-loaded and PTX-CR-6-loaded nanocapsules compared with spectrum for the empty nanocarriers. The representative peak at 262 nm provided evidence of the efficacious encapsulation of hydrophobic drug PTX in polymeric nanocarriers, also reported by Karabasz and co-workers [10]. The studied dyes show single absorption band maximum around 460 and 538 nm for CR-6 and NR, respectively in water dispersions of nanocapsules, whereas it is slightly red shifted compared with fluorescent markers dissolved in another solvents described in literature [30, 31].

The fluorescence emission spectra of NR or CR-6 co-encapsulated with PTX in the polymeric nanocarriers were measured at excitation wavelengths of *λ*_exc_ 491 nm and *λ*_exc_ 458 nm, respectively. Figure 2 shows emission spectra of the loaded compounds and the dyes dissolved in tetrahydrofuran (THF). From the spectra, we observe blue shifts for the both fluorescent markers (Δ*λ*_NR_ = 12 nm and Δ*λ*_CR-6_ = 5 nm) resulting probably from the change in solvent environment of NR and CR-6 due to their encapsulation in the polymeric nanocarriers. Encapsulation itself produced blue shifts of the emission maximum, which were observed also by other researchers [32] and can be explained via an effect of solvent orientational relaxation [31].

The fluorescence properties of nanocapsule water dispersions were investigated also by CLSM. Figure 2 shows CLSM images of the palm oil nanocapsules loaded by PTX + NR or PTX + CR-6, which examples were taken after excitation at 559 and 456 nm, respectively. It can be seen that NR and CR-6, red and green fluorescent markers, were successfully encapsulated into polymeric nanocarriers without detecting any punctuate red and green signals of aggregated nanocapsules. The most significant differences in encapsulation efficacy were observed in the polymer and oil types used for nanocapsule fabrication. The most effective co-encapsulation results were obtained for system 1c (PTX 80 %, NR 89 %) and for system 3c (PTX 76 %, CR-6 84 %). As can be seen from Table 2, the drug encapsulation efficiency of the nanocapsules stabilized by PLGA with Capmul MCM 10 oil core was higher than the PLA and PCL nanocarriers, suggesting the stronger binding affinity between hydrophobic PTX and PLGA polymer. This observation is comparable to the results reported for other polymeric nanocarriers [29, 33], which indicates the capably encapsulated hydrophobic compounds with high efficiency for anticancer applications. Furthermore, the Capmul MCM 10 has a lower hydrophilic–lipophilic balance (HLB approximately 5–6), than palm oil (HLB 10) and coconut oil (HLB 8); therefore, the nanocapsules with its oleic core may have better properties to binding PTX molecules as other drug delivery systems of highly hydrophobic compounds [34].

**Fig. 3:**
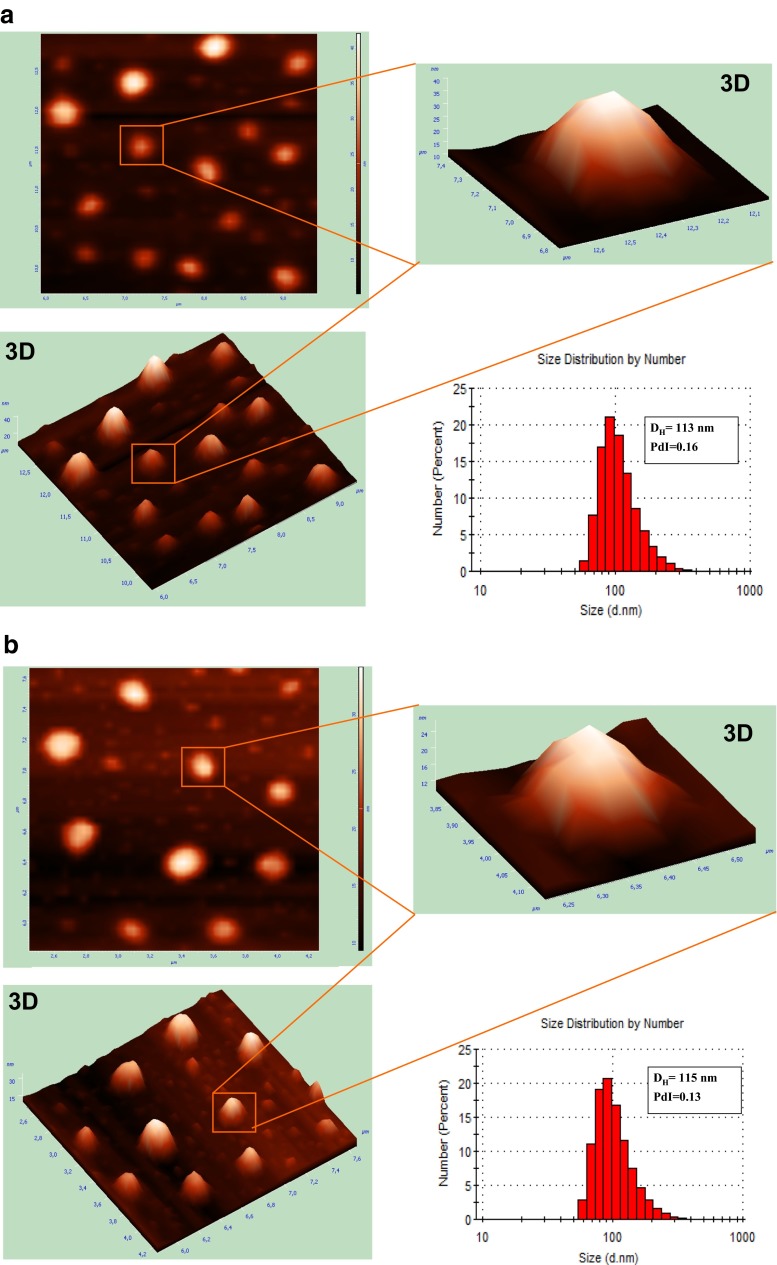
**a** AFM images on the example of PTX- and NR-loaded in Cremophor EL/PLA/coconut oil nanocapsules compared to particle size measurement by DLS. **b** AFM images on the example of PTX- and CR-6-loaded in Cremophor EL/PLA/coconut oil nanocapsules compared to particle size measurement by DLS

The appropriate morphology of nanocarriers is crucial in the designing of drug delivery systems used in diagnosis and therapy. AFM is a technique, with dimensional resolutions from 0.1 up to 10 nm, that offers a unique chance for visualizing nanoparticles in natural surroundings [35]. Furthermore, it allows direct measurement of size in dried state of probes, which lets contemporary characterization of nanoconstructs’ shape and structure. The microscopic technique in tapping mode was used to confirm that there was no tendency to aggregation or adhesion among obtained nanocapsules. The AFM 3D image of an individual polymeric nanocapsule in Fig. 3a shows its semi-smooth surface without certain roughness, although 2D images display relatively smoother surface morphology. Figure 3b also reveals that the nanocapsules were semi-spherical in shape and of size near 200 nm. Therefore, AFM investigated the nanocapsule sizes, which are in the good agreement with the hydrodynamic diameters predicted from DLS. The similar results of size measurements were obtained previously [36] in relation to morphology of PTX encapsulated in PLGA nanoparticles examined by AFM method.

Besides, PTX, NR, and CR-6 compounds showed no significant influence on the zeta potential of the polymeric formulations. It is known that measurement of *ζ*-potential is important to predict the stability of colloidal dispersions or polymeric nanocapsules in water solutions. In general, particle aggregation is less likely to occur for highly charged particles (with high zeta potential values) because the repulsion forces arising from the surface charge can overcome the Van der Waals attractive forces between the particles. Ordinarily, an absolute zeta potential value of 20 mV or higher is indicative of a stable system [18, 23]. However, this rule cannot be strictly applied for systems which contain steric stabilizers (e.g., surfactants or polymers with high HLB value), because its adsorption will decrease the zeta potential due the shift in the shear plane of the particle [21]. As depicted in Table 2, results of zeta potential measurements showed that the surface charges of all polymeric nanocapsules prepared in various polymer/oil compositions were consistently negative. All systems revealed a *ζ*-potential of about −1 to −6 mV at fixed nonionic emulsifier concentration of 1 % (*w*/*w*), and no effect of the liquid core (different oil types) and polymeric shell (various polyesters) on the surface charge was observed. The lower or comparable results of *ζ*-potential were obtained by other authors, which prepared also polymeric nanoparticles loaded with PTX but with different outer surface (PEG, WGA conjugated) and concentration of polymers [33, 37]. The *ζ*-potential of the PTX-NR-loaded and PTX-CR-6-loaded nanocarriers was slightly higher than that of blank nanoparticles probably due to the partial adsorption of PTX, NR, and CR-6 on the surface of nanocapsules during preparation process. Thus, similar interface properties can be assumed for all nanocapsules, suggesting that molecules of Cremophor EL^®^ probably stabilize interface of studied systems by steric repulsions of the Stern layer [38]. Furthermore, as it has been describe previously, neutrally charged particles have a much lower opsonization rate (followed by macrophages clearance) than those with high surface charge. Therefore, the well-designed nanocarriers for drug delivery should have a surface charge either slightly positive or slightly negative for long-term circulation in the blood stream, which requirement being well satisfied by our study results [39].

**Fig. 4:**
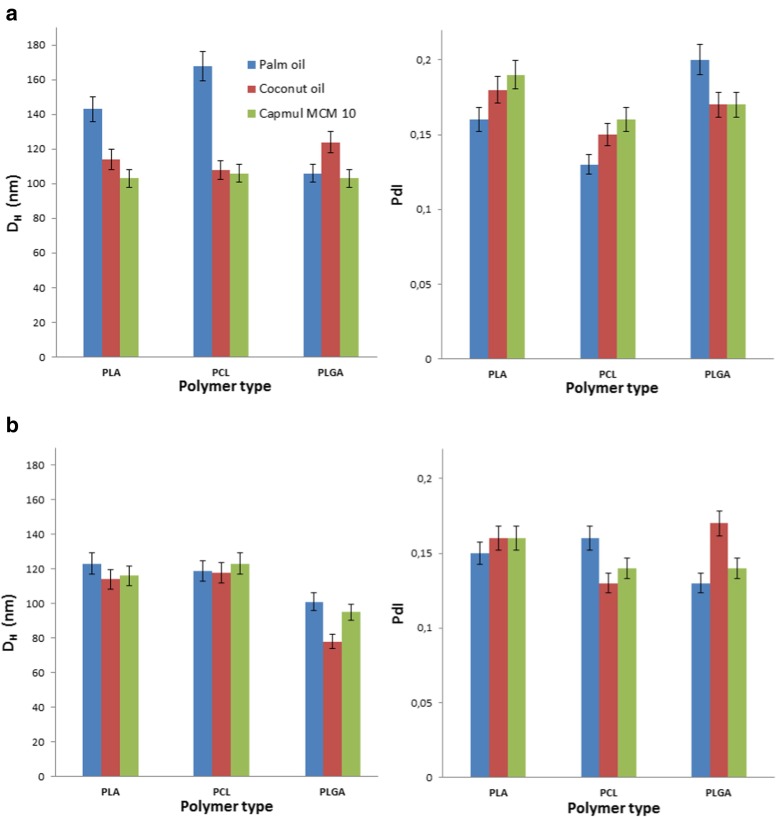
Variations in size and polydispersity of nanocarriers after storage in the dark (*T* = 40 days); **a** PTX-NR-loaded nanocapsules and **b** PTX-CR-6-loaded nanocapsules

The assessment of particle size and particle size distribution (Fig. 4, Table 1) of PTX-CR-6- and PTX-NR-loaded nanocapsules displayed good stability properties upon storage for 40 days. All systems showed only slight increase in *D*_H_ and PdI (between 1 and 5 %) values. In contrast, some PTX formulations described in literature [12, 40] were stable for about 2–3 weeks only; then, a distinct increase in particle size and particle size distribution was observed. Systems 1c, 2c, and 3c, with Capmul MCM C10 oleic core (both, loaded, and empty), showed a slightly smaller mean diameter and also higher stability in the period of time, than nanocapsules with palm and coconut oleic cores (and Fig. 4). The observed phenomenon could indicate those nanocapsules as potential drug delivery systems for cellular internalization and theranostic application. However, all nanosystems with hydrophobic fluorescent dyes show high photochemical stability and fluorescence properties for at least 40 days.

### In vitro localization of loaded nanocarriers in human tumor cells

**Fig. 5:**
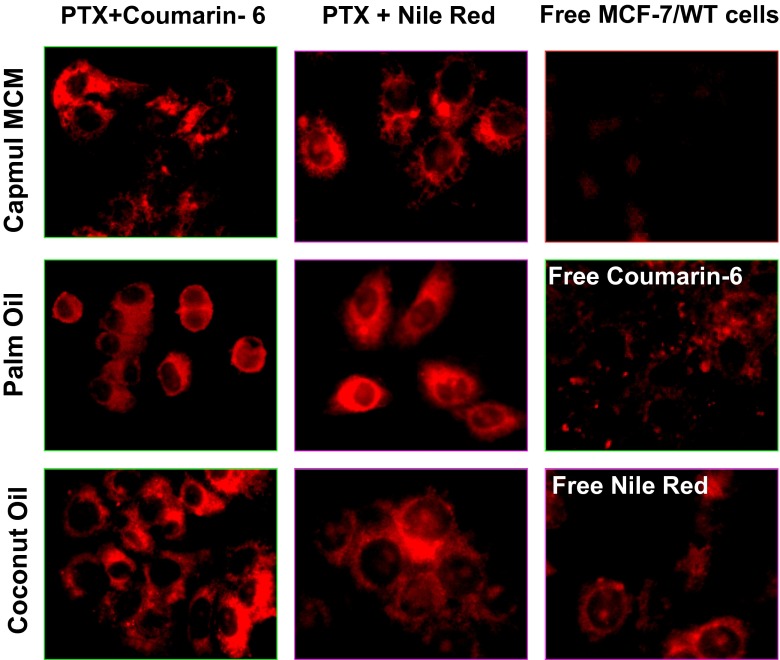
Cellular localization of PTX-loaded nanocapsules stabilized by PLA and labeled by coumarin-6 or Nile Red in relation to the nonloaded fluorescent marker molecules (positive control) and untreated MCF-7/WT cancer cells (negative control)

Owing to their fluorescent properties, CR-6 and NR are among the best luminescent markers for cell labeling and multiplexed imaging [12, 26] including CLSM—one of the most common imaging techniques allowing one to obtain high-resolution optical images with depth selectivity. CLSM is useful for imaging cells labeled by markers such as CR-6 and NR and is extensively used in numerous biological science disciplines [10, 12, 15, 25]. As part of the characterization of PTX-CR-6- and PTX-NR-loaded nanocapsules, CLSM images were taken to record the uptake and the intracellular free and encapsulated fluorescent marker distribution in the cell monolayer of microcultures of human breast MCF-7/WT cells. After 24 h of the cell incubation with PTX nanocarriers labeled by a fluorescence marker, CR-6 or NR molecules were regularly diffused in the cell cytoplasm with significantly higher fluorescence around the nucleus envelope (Fig. 5). The presence of both loaded polyester nanocapsules did not change the cells morphology, which corresponded with good metabolic conditions of MCF-7/WT culture. The insignificant autofluorescence of the untreated MCF-7/WT control cells was observed in the control CLSM experiments. Moreover, the presence of empty nanocapsules not influenced of the MCF-7/WT autofluorescence (data not show). Additionally, the cell morphology changes were not remarkable, indicating that the nanocapsules without PTX did not have influence on breast cancer cell metabolic conditions. From Fig. 5, we can observe slightly improved cellular internalization of nanocapsules with oleic core composed of palm oil and coconut oil in comparison to those with Capmul MCM. However, the fluorescence emission of an excited NR or CR-6 observed in CLSM experiments indicates that the studied dyes essentially do not aggregate inside the cells and can, therefore, act as efficient fluorescent markers for theranostic application, although the results of our in vitro experiments indicated that the free NR or CR-6 molecules were less effectively taken up by MCF-7/WT cells in comparison to the encapsulated dye molecules. Furthermore, it is worth noting that free dye molecules could also be absorbed in vivo by regular cells of the surrounding healthy tissue. The encapsulation of fluorescent markers makes it possible to offer more advantages in comparison to free photo-dependent active agent as a consequence of preventing the delocalization of its molecules and overcoming serious side effects after light excitation.

## Conclusions

We have presented a low-cost and high-throughput procedure for the fabrication of stable, near-monodisperse, and biocompatible drug delivery systems on a nanoscale. The fabricated polyester-based nanocapsules, primary with PLGA shell, show high encapsulation efficiency, long-term physical stability, and improved cellular internalization of multifunctional cargo. Our results provide conclusions that the obtained nanocapsules might become useful as efficient drug delivery systems of poorly water-soluble cytostatics as PTX, dedicated for theranostic application. We also proved that the oil core nanosystems could be used as a promising alternative to the common nanocarriers, presenting considerable perspective for application in potential diagnostics and chemotherapy of breast cancer.
